# Ultrasound imaging of the axilla

**DOI:** 10.1186/s13244-023-01430-9

**Published:** 2023-05-11

**Authors:** Giulio Cocco, Vincenzo Ricci, Costantino Ricci, Ondřej Naňka, Orlando Catalano, Antonio Corvino, Andrea Boccatonda, Francesco Lorenzo Serafini, Jacopo Izzi, Gianfranco Vallone, Vito Cantisani, Giovanni Iannetti, Massimo Caulo, Claudio Ucciferri, Jacopo Vecchiet, Andrea Delli Pizzi

**Affiliations:** 1 Department of Neuroscience, Imaging and Clinical Sciences, G. d’Annunzio University, Chieti, Italy; 2 Unit of Ultrasound in Internal Medicine, Department of Medicine and Science of Aging, G. d’Annunzio University, Chieti, Italy; 3 Physical and Rehabilitation Medicine Unit, Luigi Sacco University Hospital, ASST Fatebenefratelli-Sacco, Milan, Italy; 4 Department of Experimental, Diagnostic and Specialty Medicine (DIMES), University of Bologna, Bologna, Italy; 5 First Faculty of Medicine, Institute of Anatomy, Charles University, Prague, Czech Republic; 6 Department of Radiology, Istituto Diagnostico Varelli, Naples, Italy; 7 Movement Sciences and Wellbeing Department, University of Naples Parthenope, Naples, Italy; 8 Internal Medicine, Bentivoglio Hospital, AUSL Bologna, Bentivoglio, Italy; 9 Unit of Radiology, Santissima Annunziata Hospital, Chieti, Italy; 10 Department Life and Health V. Tiberio, Università Degli Studi del Molise, Campobasso, Italy; 11 Department of Radiology, Oncology, Anatomo-Pathology, Sapienza-University of Rome, Rome, Italy; 12 Ospedale S. Spirito, Università Degli Studi Chieti-Pescara, Chieti, Italy; 13 Clinic of Infectious Diseases, Department of Medicine and Science of Aging, G. d’Annunzio University, Chieti, Italy; 14 Department of Innovative Technologies in Medicine and Dentistry, University G. d’Annunzio, Chieti, Italy

**Keywords:** Ultrasound, Axilla, Histopathology

## Abstract

**Abstract:**

Axilla is a pyramidal-in-shape “virtual cavity” housing multiple anatomical structures and connecting the upper limb with the trunk. To the best of our knowledge, in the pertinent literature, a detailed sonographic protocol to comprehensively assess the axillary region in daily practice is lacking. In this sense, the authors have briefly described the anatomical architecture of the axilla—also using cadaveric specimens—to propose a layer-by-layer sonographic approach to this challenging district. The most common sonographic pathological findings—for each and every anatomical compartment of the axilla—have been accurately reported and compared with the corresponding histopathological features. This ultrasound approach could be considered a ready-to-use educational guidance for the assessment of the axillary region.

**Critical relevance statement:**

Axilla is a pyramidal-in-shape “virtual cavity” housing multiple anatomical structures and connecting the upper limb with the trunk. The aim of this review article was to describe the anatomical architecture of the axilla, also using cadaveric specimens, in order to propose a layer-by-layer sonographic approach to this challenging district.

**Graphical abstract:**

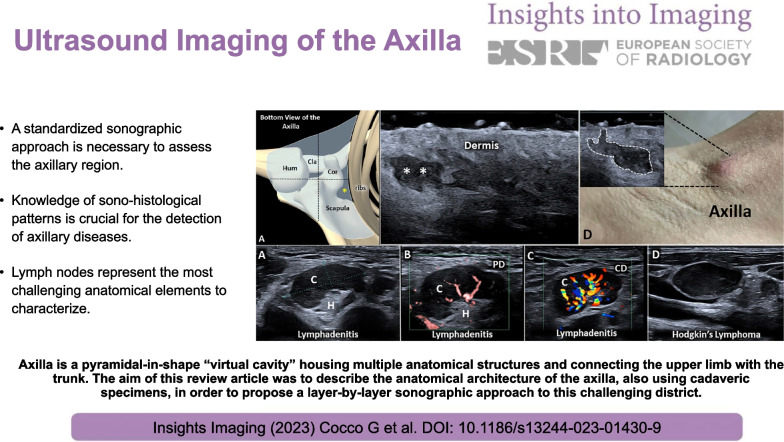

**Supplementary Information:**

The online version contains supplementary material available at 10.1186/s13244-023-01430-9.

## Key points

- A standardized sonographic approach is necessary to assess the axillary region.
- Knowledge of sono-histological patterns is crucial for the detection of axillary diseases.
- Lymph nodes represent the most challenging anatomical elements to characterize.

**Supplementary Information:**

The online version contains supplementary material available at 10.1186/s13244-023-01430-9.

## Introduction

Ultrasound imaging is widely used in daily practice to assess the musculoskeletal system for both upper and lower limbs. Muscles and tendons, peripheral nerves, joints and superficial soft tissues are the anatomical structures most commonly evaluated [1–4]. The transitional zones between the limbs and the trunk present a very complex anatomical architecture, but, unlike the inguinal region, a standardized and comprehensive sonographic protocol to assess the axilla is lacking in the recent literature [5–7]. In this sense, firstly we briefly reviewed the pertinent literature about the anatomy of the axillary region using cadaveric specimens to accurately localize the different histo-anatomical compartments [8, 9]. Then, starting from a comprehensive knowledge of the basic anatomy, we proposed an easy-to-use sonographic approach to the axillary region describing several pathological conditions usually encountered in clinical practice. A standardized sonographic protocol is necessary to provide new insights and accurately evaluate a challenging anatomical site as the axilla in daily practice.

### Anatomy of the axillary region

**Fig. 1:**
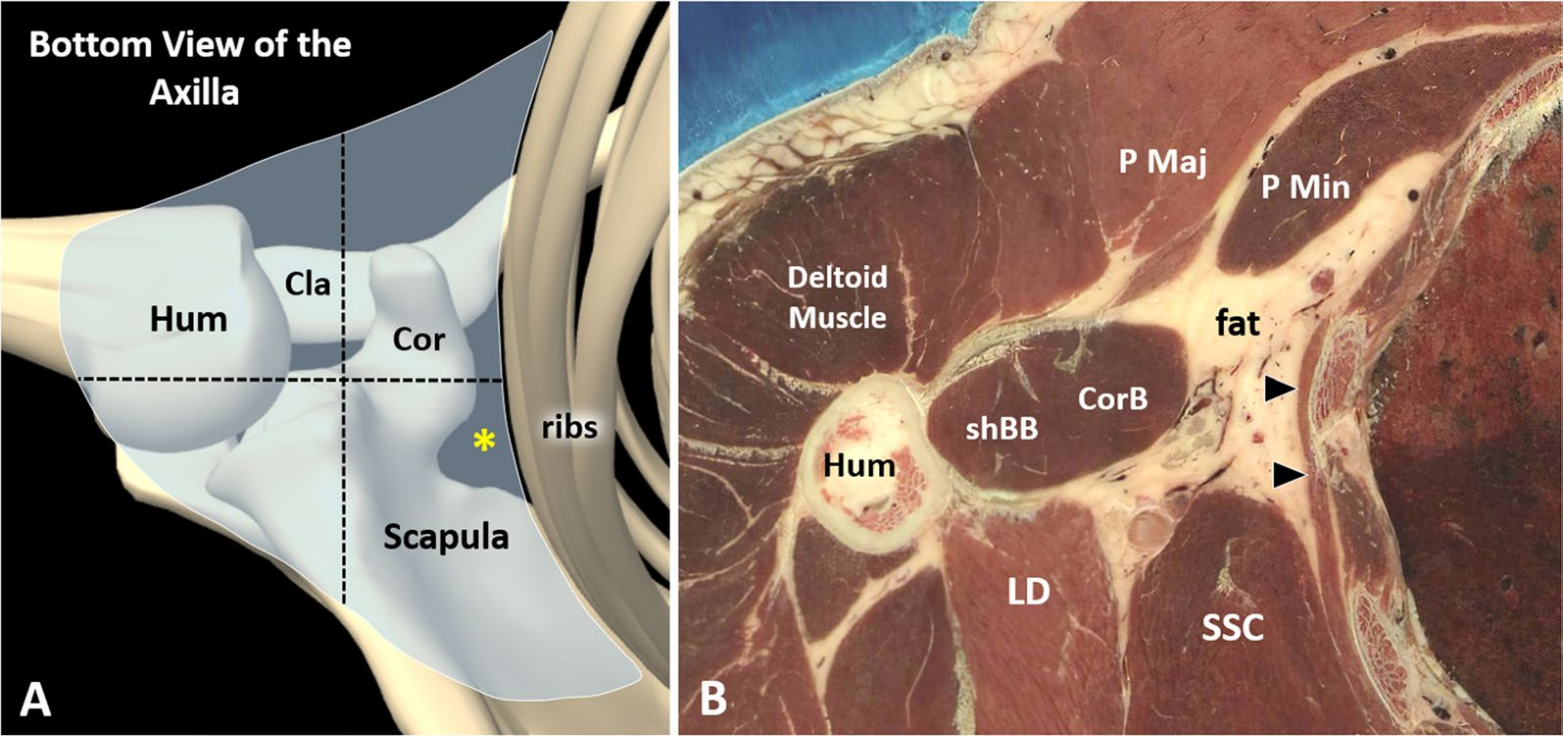
From bony to muscular edges of the axillary region. The axilla is an irregular-in-shape anatomical region *(light blue)* located between the ribs, the scapula, the clavicle *(Cla)* and the proximal humerus *(Hum)* (**A**). The pectoralis major *(P Maj)* and minor *(P Min)* muscle in the anterior compartment, the coracobrachialis *(Cor B)* and short head of the biceps brachii muscle *(shBB)* in the lateral section, the serratus anterior muscle *(black arrowheads)* medially; and the subscapularis muscle *(SSC)*, the teres major and latissimus dorsi muscle *(LD)* in the posterior compartment, can be considered the “soft” anatomical landmarks to better define this complex anatomical space (**B**). Cor: coracoid; yellow asterisk: cervicoaxillary canal; black dotted lines: quadrants

**Fig. 2:**
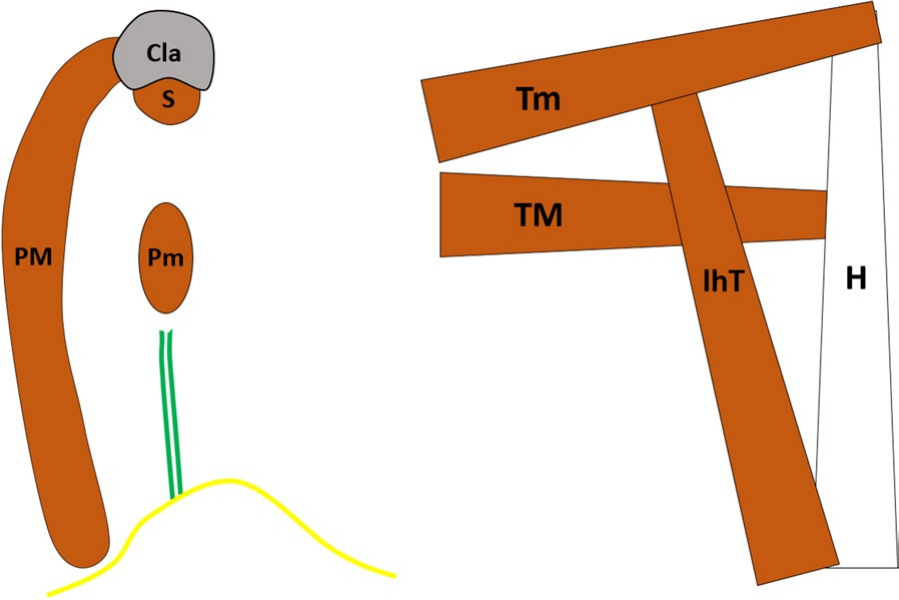
Schematic drawings of the axillary anatomical elements. The sagittal view (**A**) shows how the clavipectoral fascia *(white line)* wraps the pectoralis major *(PM)* muscle, the pectoralis minor *(Pm)* muscle and the subclavius *(S)* muscle; and blends with the axillary fascia *(yellow line)* and the suspensory ligament of the axilla *(green lines)*. Likewise, the posterior view (**B**) shows the quadrilateral space *(QS)*, the triangular space *(TS)* and the triangular interval *(TI)* originating by the crossing of the teres minor *(Tm)* muscle, the teres major *(TM)* muscle, the long head of the triceps *(lhT)* muscle and the humerus *(H)*. Cla: clavicle

Axilla is an anatomical region located at the junction of the upper limb with the thorax. It presents a pyramidal shape with four walls—anterior, posterior, medial and lateral—an open apex and a large base. Pectoralis major muscle, pectoralis minor muscle and subclavius muscle define the anterior edge; subscapularis muscle, teres major muscle and latissimus dorsi muscle constitute the posterior wall; ribs, serratus anterior muscle and intercostal muscles define the medial border; and coracobrachialis muscle and short head of the biceps muscle are the lateral boundaries (Fig. 1) [8, 9]. Of note, the clavipectoral fascia connects the pectoralis major muscle, the pectoralis minor muscle and the subclavius muscle and presents a histological continuum with the suspensory ligament of the axilla and the axillary fascia (Fig. 2). All the aforementioned fascial elements guarantee the concave shape of the axillary base. Clavipectoral fascia is normally pierced by the lateral pectoral nerve, the thoracoacromial vessels and the cephalic vein [8, 9]. The open apex of the axilla is called cervicoaxillary canal and represents the anatomical connection between the axillary region and the supraclavicular fossa. It is bordered anteriorly from the clavicle, medially from the first rib, laterally from the coracoid process of the scapula (Fig. 1); and it is crossed by subclavian vessels and the brachial plexus [8, 9]. Axillary fascia travels from the inferior edge of the pectoralis major muscle anteriorly to the inferior edge of the latissimus dorsi muscle posteriorly, defining the base of the axilla and merging with the brachial fascia laterally [8, 9]. Quadrangular space (or quadrilateral space) is a gap in the posterior wall of the axilla bordered by the shaft of the humerus laterally, the long head of the triceps medially, the teres minor muscle superiorly and the teres major muscle inferiorly (Fig. 2) [10]. The axillary nerve and the posterior circumflex humeral artery—a branch of the axillary artery—normally cross the quadrilateral space passing beneath the inferior edge of the subscapularis muscle and wrapping from anterior to posterior the neck of the humerus (i.e., the circumflex nerve) [11]. Indeed, the posterior cord of the brachial plexus splits inferiorly to the glenohumeral joint giving rise to the axillary nerve that courses within the axilla posteriorly to the axillary artery and anteriorly to the subscapularis muscle to innervate distally—with its motor fibers—the deltoid and teres minor.

**Fig. 3:**
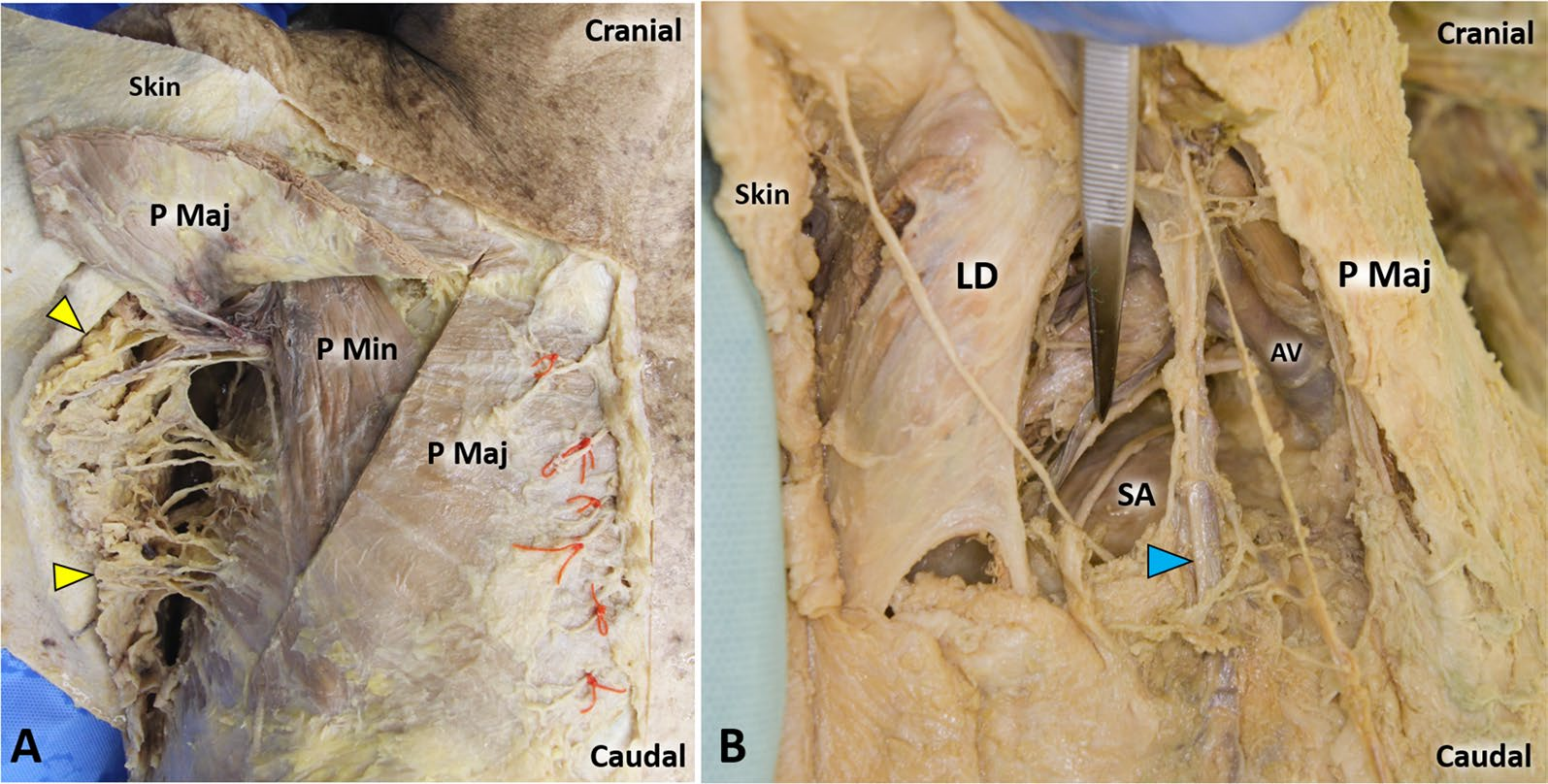
Cadaveric anatomy of the axillary region. Using an anterior approach (**A**) and lifting the skin and pectoralis major muscle (P Maj), the axillary fat tissue *(yellow arrowheads)* can be easily observed filling the anatomical space between the upper limb and the trunk. Using an inferior approach **B** and selectively removing the fat pad, a panoramic view of the axillary cavity can be acquired until its medial border—i.e., the serratus anterior *(SA)* muscle. Of note, the more superficial vein is the lateral thoracic vein *(light blue arrowhead)*; instead, the axillary vein *(AV)* is located in the deep portion of the axilla (**B**). P Min: pectoralis minor muscle; LD: latissimus dorsi muscle

The main anatomical elements located within the axillary space are the axillary artery and vein, brachial plexus, lymph nodes/lymphatic network, fat tissue, accessory breast tissue, skin and adnexal structures (Fig. 3). Accessory breast tissue, also known as ectopic breast tissue, is a residual tissue that persists from normal embryological development and can be found in up to 6% of the population [12]. The spatial distribution of axillary lymph nodes is highly variable in the general population, but two main levels are commonly distinguished in relation to their depth (i) epifascial lymph nodes—located superficially to the axillary fascia—and (ii) deep lymph nodes, which usually surround the axillary neurovascular bundle (e.g., apical and lateral lymph nodes) [8].

### Scanning technique

The patient is commonly positioned supine with the upper limb abducted and externally rotated to optimally expose the axillary surface. Sometimes a pillow can be used in between the examination bed and scapula to lift the axilla from the support surface, optimizing the scanning of its more posterior section [13]. Of note, to evaluate/quantify the articular effusion of the shoulder using the axillary approach, the standing position is preferred taking advantage of gravitational force and shifting fluid toward the axillary recess of the glenohumeral capsule. High-frequency ultrasound probes are commonly used to assess the content of the axilla considering the high spatial resolution ideal to visualize superficial anatomical structures. Broadband linear probes allow reducing the frequency in patients with high body mass index and/or large amounts of axillary fat tissue to depict more deep planes [13].

Comprehensive scanning of the axillary region must be performed covering the entire space between the pectoralis major muscle anteriorly and the latissimus dorsi posteriorly (Fig. 3) [14]. In this sense, to accurately report the spatial location of pathological sonographic findings, the authors suggest considering a bottom view of the axillary region with four main quadrants: anteromedial, anterolateral, posteromedial and posterolateral quadrant (Fig. 1) [13]. Scanning all the aforementioned quadrants and using a layer-by-layer approach from superficial to deep planes (Table 1), a standardized and reproducible sonographic assessment can be guaranteed. For each and every pathological finding, two orthogonal planes in grayscale modality and color/power Doppler assessment must be coupled with the medical history and clinical scenario. Peculiar attention should be paid to the values of the pulse repetition frequency and the accurate set of the color gain (avoiding noise artifact) to precisely evaluate the perfusion pattern of pathological sonographic findings [3].

**Table 1:** Axillary region sonographic assessment checklist

Basic scanning protocol	Advanced scanning protocol
Skin and adnexal structures	Accessory breast tissue
Axillary fat tissue	Glenohumeral joint
Lymph nodes	Lymphatic collectors
Neurovascular bundle	

### Sono-anatomy of the axillary region

**Fig. 4:**
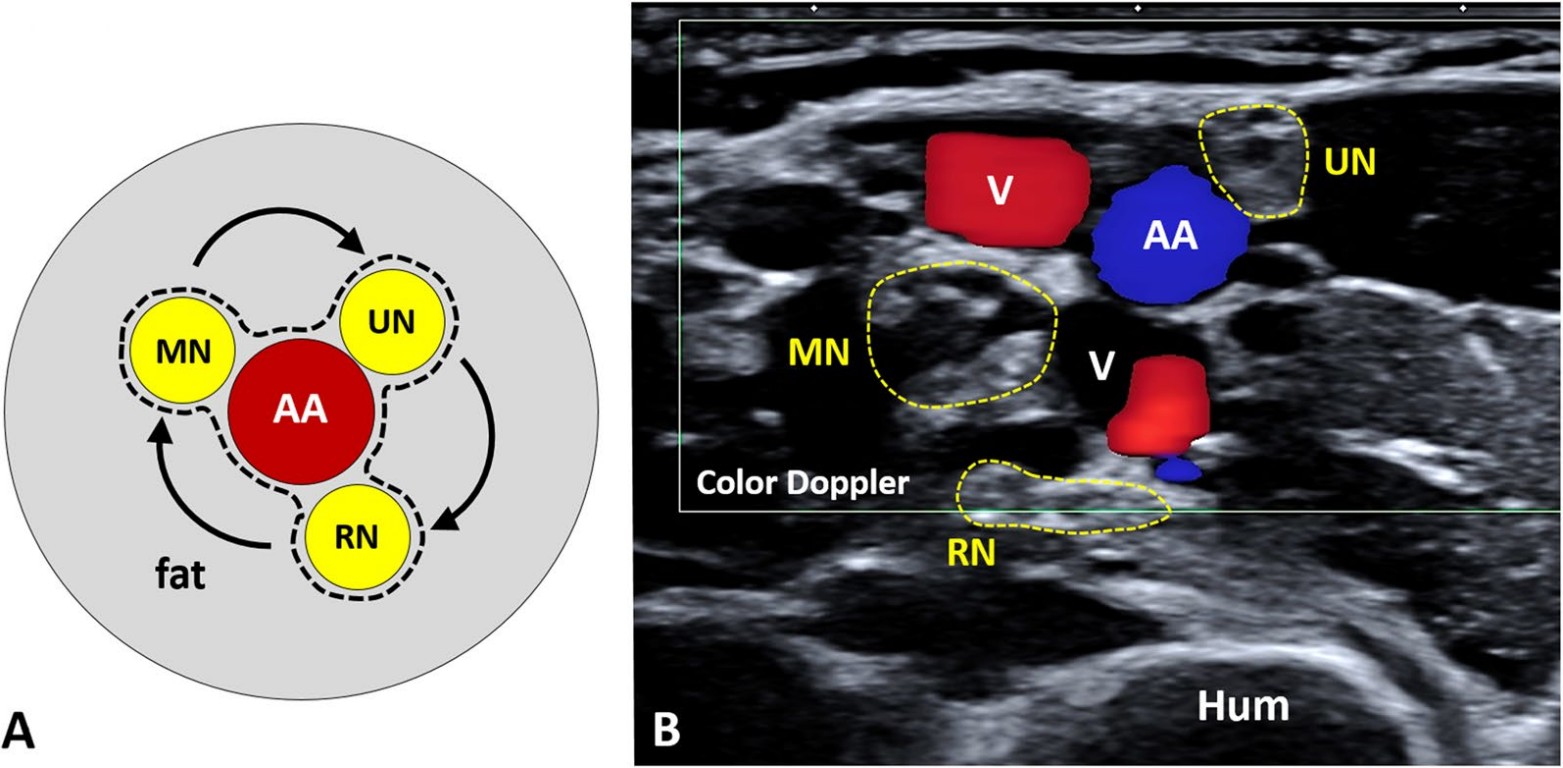
Neurovascular bundle of the axilla. The ulnar nerve *(UN)*, the radial nerve *(RN)* and the median nerve *(MN)* wrap the axillary artery *(AA)* in a clockwise fashion *(curved dark arrow)* at the axillary base (**A**). They present a typical honeycomb pattern *(yellow dotted line)* and can be easily differentiated from the vascular elements using the color/power Doppler (**B**). V: vein; Hum: humerus; black dotted line: axillary sheath

Dermo-epidermal complex is the most superficial layer of the axilla. It presents a trilaminar pattern with a superficial hyperechoic line representing the epidermis, an echoic band representing the dermis and a deep hyperechoic interface in between the dermis and subcutis (dermo-hypodermal interface) [4]. Some hypoechoic islands can be physiologically visualized within the echoic band of the dermis, especially using very high-frequency probes; they are usually related to the presence of adnexal structures—e.g., pilosebaceous unit [15]. In detail, the subcutaneous tissue and the axillary fat pad can be easily recognized with their peculiar architecture—a hyperechoic fibrous scaffold that mechanically stabilizes multiple, hypoechoic, fat lobules [4]. A transverse scan of the axillary neurovascular bundle allows the simultaneous visualization of the axillary artery and vein—anechoic rounded structures (i.e., the “black bubbles”)—and of the neural elements of the brachial plexus with a typical honeycomb pattern [3]. In this sense, the ulnar nerve, the radial nerve and the median nerve surround the axillary artery clockwise, starting from the superomedial quadrant of the circular axillary base (Fig. 4). Interestingly, the entire neurovascular bundle is enclosed within a fibrous sheath—known as the axillary sheath and surrounded by fat tissue—which represents an extension of the prevertebral layer of the deep cervical fascia. Proximal-to-distal or vice versa sonographic tracking is commonly performed to panoramically evaluate the vascular/neural elements along their course, and the longitudinal scan must be always coupled with the transverse scans, especially in the case of suspected pathology. Color/power Doppler can be promptly used to confirm the exact location of the main vascular elements and their smaller branches if clinically indicated.

In normal conditions, lymph nodes appear as flattened and hypoechoic anatomical structures. Chronic degenerative changes can progressively lead to a benign replacement of the lymphoid tissue with fat, starting from the inner portion and progressively involving the cortical section. In this sense, high reflective defects within lymph nodes should be always cautiously interpreted, by integrating grayscale findings, the perfusion pattern and the clinical scenario [16].

Of note, positioning the probe in a longitudinal plane, the axillary pouch of the glenohumeral joint can be easily observed as a thin hyperechoic band connecting the inferior labrum with the proximal humerus. Rotating the probe of 90 degrees (transverse plane) and centering the humeral head, two focal thickenings of the inferior shoulder capsule can be recognized—the anterior and posterior bands of the inferior glenohumeral ligament [17].

## Sonographic pathological findings

### Skin and adnexal structures

At the axilla, the most common pathologies involving the skin and adnexal structures in daily practice are seborrheic keratosis, epidermal inclusion cyst, hidradenitis suppurativa and pseudofolliculitis cutis. Seborrheic keratosis refers to a knobby overgrowth of keratinocytes with a warty appearance, and it can be considered a benign skin lesion [14].

**Fig. 5:**
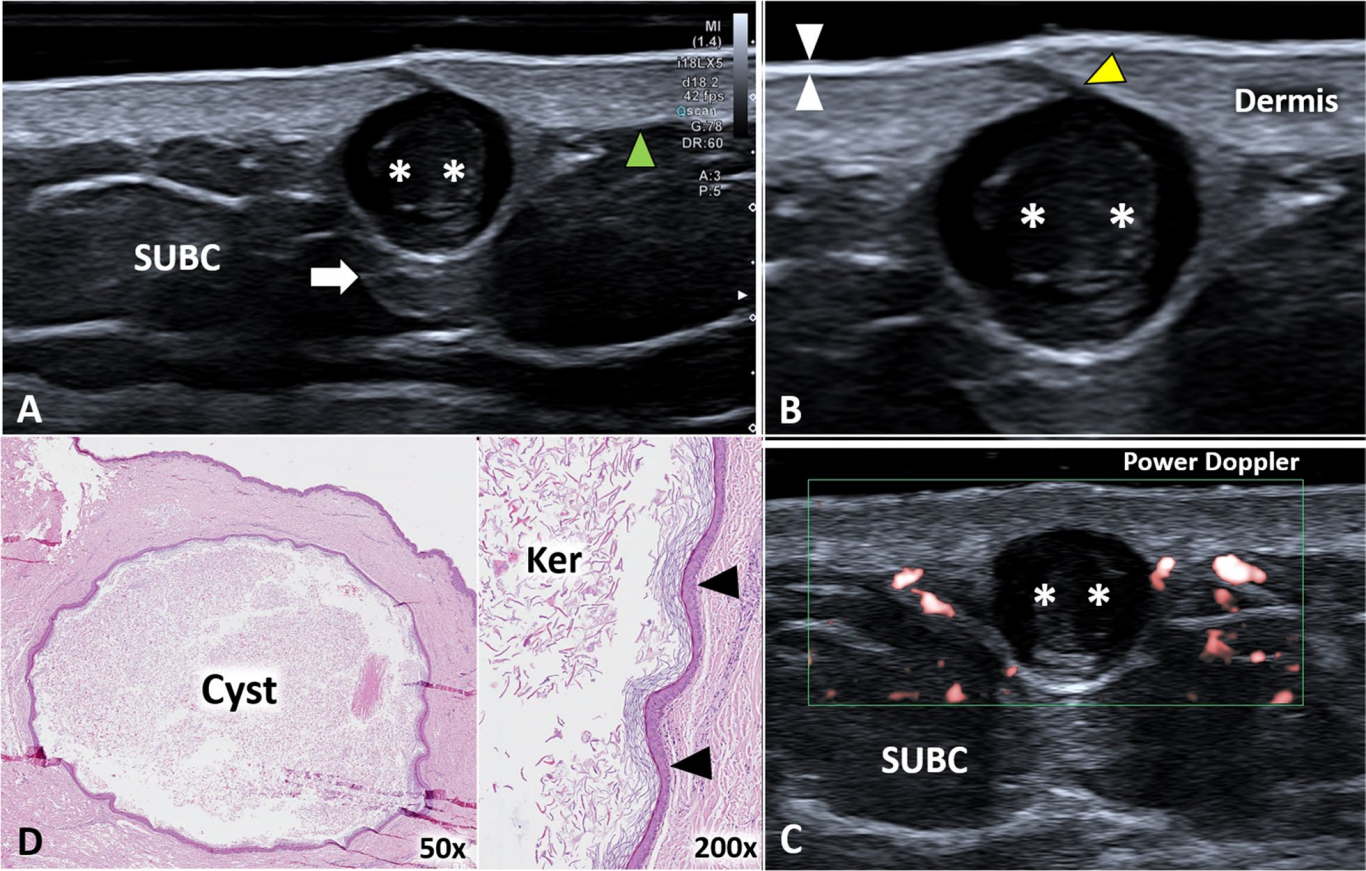
Epidermal inclusion cyst of the axilla. A hypoechoic and rounded formation *(white asterisks)* can be easily observed originating from the dermis, crossing the dermo-hypodermal interface *(green arrowhead)* and protruding within the subcutaneous tissue *(SUBC)* with a typical artifact of posterior acoustic enhancement *(white arrow)* (**A**). Of note, using high-frequency probes, the canaliculus *(yellow arrowhead)* between the cyst *(white asterisks)* and epidermis *(white arrowheads)* can be clearly recognized (**B**). High-sensitivity color Doppler does not show intra-cystic vascular signals but only some small vessels in the surrounding soft tissues (**C**). The histological examination confirms the presence of the unilocular and spherical cyst (**D**) lined by a multi-layered epithelium *(black arrowheads)* and containing laminated keratin *(Ker)*. H&E: hematoxylin & eosin

Epidermal cysts are benign neoformation—presenting a cellular wall and an inner cavity filled with keratin—usually located within the dermis and/or subcutaneous tissue [15]. On ultrasound imaging, they appear as hypo/anechoic rounded elements with a typical artifact of posterior acoustic enhancement and well-defined edges (Additional file 1: Video 1) [18, 19]. In some cases, a thin hypoechoic canaliculus can be identified, connecting the cyst with the overlying epidermis—i.e., the punctum (Fig. 5). Several triggers can lead to progressive dilatation of the epidermal cyst until the partial/total rupture with the spreading of keratin and inflammatory fluids within the surrounding soft tissues. No microvessels are usually identified within the intact epidermal cyst, but abnormal hypervascularization can involve the peri-cystic tissues in case of inflammatory conditions (Fig. 5) [19, 20]. In case of rupture, granulation tissue can migrate within the lumen of the cyst; in this sense, intracystic vascular signals during the color/power Doppler assessment can be considered an indirect sign of cystic wall damage [20].

**Fig. 6:**
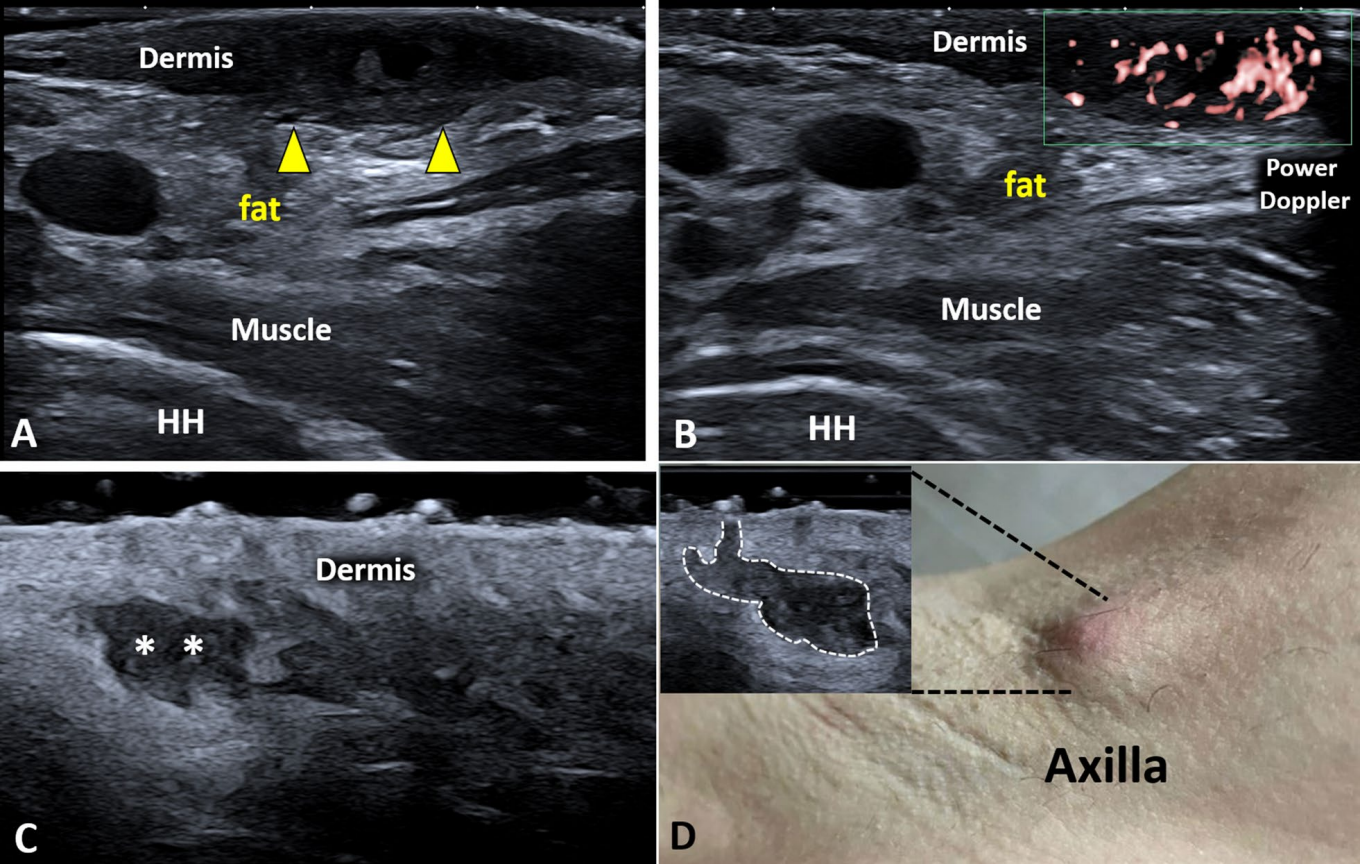
Hidradenitis suppurativa of the axilla. Massive hypoechoic thickening *(yellow arrowheads)* of the dermis (**A**) with pathological hypervascularization on high-sensitivity color Doppler (**B**) can be promptly observed at the level of the axillary skin. Very high-frequency ultrasound probes (**C, D**) better define additional sonographic signs of hidradenitis suppurativa as the superficial, saclike fluid collections *(white asterisks)* and the ballooning of hair follicles *(white dotted line)*. *HH* Humeral head

Hidradenitis suppurativa is an immune/inflammatory disorder characterized by early lymphocytic inflammation and hyperkeratosis of the pilosebaceous unit with a progressive evolution to hair-follicle destruction and granuloma formation [21]. The main sonographic findings (Fig. 6) are dermal hypoechoic thickening, widening of the hair follicles, dermal/hypodermal pseudocysts (Additional file 2: Video 2), saclike fluid collections within the dermis and/or hypodermis (Additional file 3: Video 3) and band-like elements (also called tunnels or fistulous tracts) [19]. According to the literature, three or more of the aforementioned sonographic signs must be identified to diagnose the hidradenitis suppurativa [22]. Color/power Doppler plays a pivotal role in monitoring the vascular signals of the dermis/hypodermis and the activity of the disease (Additional file 4: Video 4) [15, 23].

**Fig. 7:**
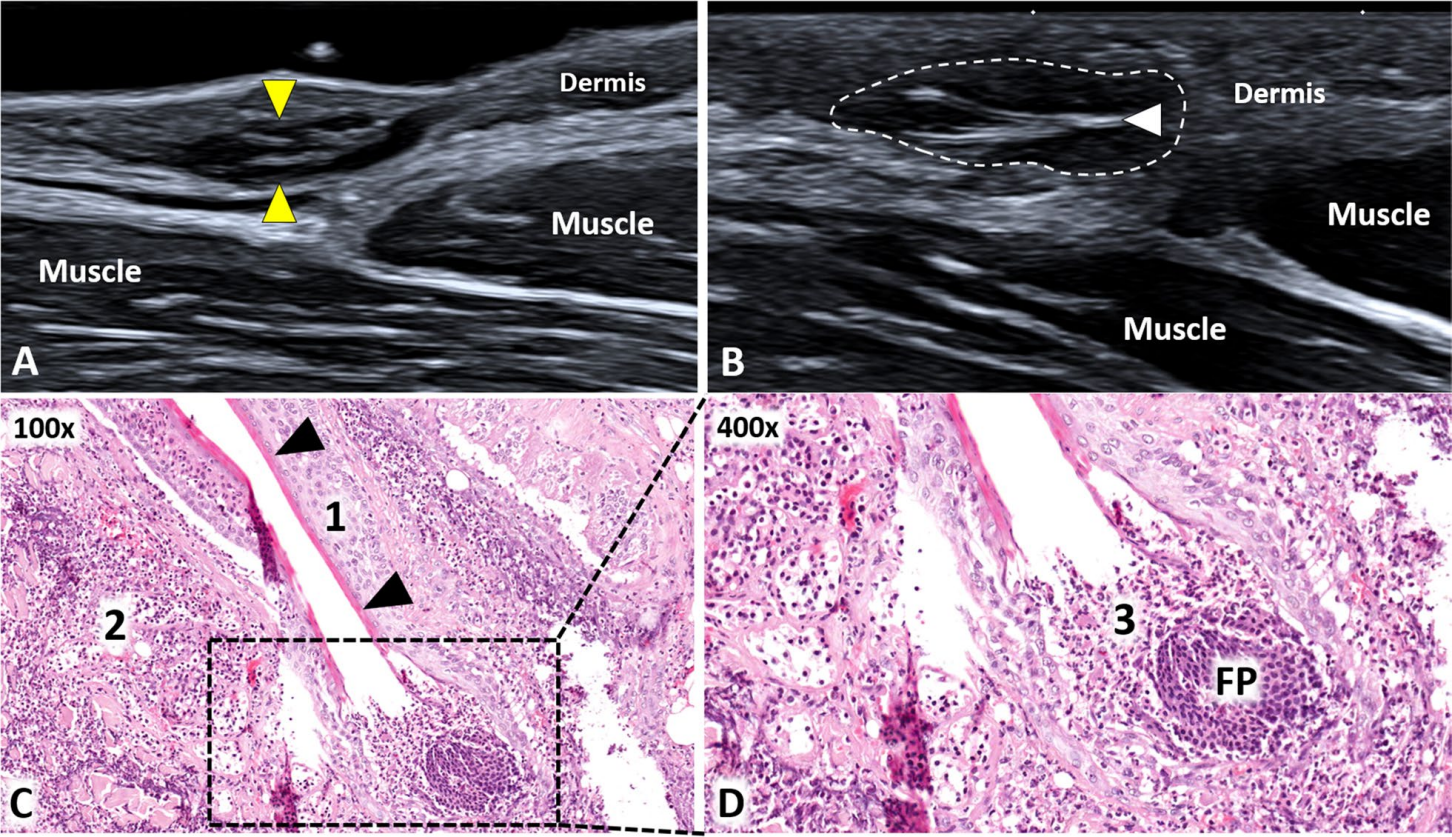
Pseudofolliculitis cutis of the axilla. Avoiding an excessive squeeze of the superficial soft tissues, also a small hypoechoic “mass” *(yellow arrowheads)* can be easily identified within the dermal layer of the axilla (**A**). Of note, a gentle sono-palpation (**B**) can be performed to promptly confirm the solid texture of inflammatory tissues *(white dotted line)* wrapping the hyperechoic “ingrown” hairs *(white arrowhead)*. **C–D** The histological examination (**C**) confirms the simultaneous presence of an acute inflammatory infiltrate (1) with neutrophilic and eosinophilic granulocytes reacting against the epithelium of the follicular structure *(black arrowheads)*—external and internal root sheath—and a chronic inflammatory infiltrate (2) with lymphocytes, plasma cells and macrophages reacting against broken hair shafts and generating foreign body giant cell reaction in the peri-follicular area. Of note, inflammatory cells (3) are also leading to a progressive disruption of the follicular papilla *(FP)* (**D**). H&E: hematoxylin & eosin

Pseudofolliculitis cutis is a chronic inflammatory disease triggered by an aberrant growth of hairs. Histologically, it usually presents dermal inflammatory changes with neutrophilic infiltration, dermal microabscesses and foreign-body giant-cell reaction surrounding the invading hair [24]. On ultrasound, an intradermal hypoechoic lesion can be easily identified with a rounded or oval shape (Additional file 5: Video 5). Gentle movements of the probe can be performed to identify the hyperechoic hair located within the reactive granulation tissue (Fig. 7). If not early diagnosed and treated, the aforementioned condition can progressively lead to scarring processes until the development of keloid [25].

### Axillary fat tissue

At the axilla, the most common pathologies involving the fat tissue in daily practice are subcutaneous abscesses, hematoma, lymphedema and fat necrosis.

**Fig. 8:**
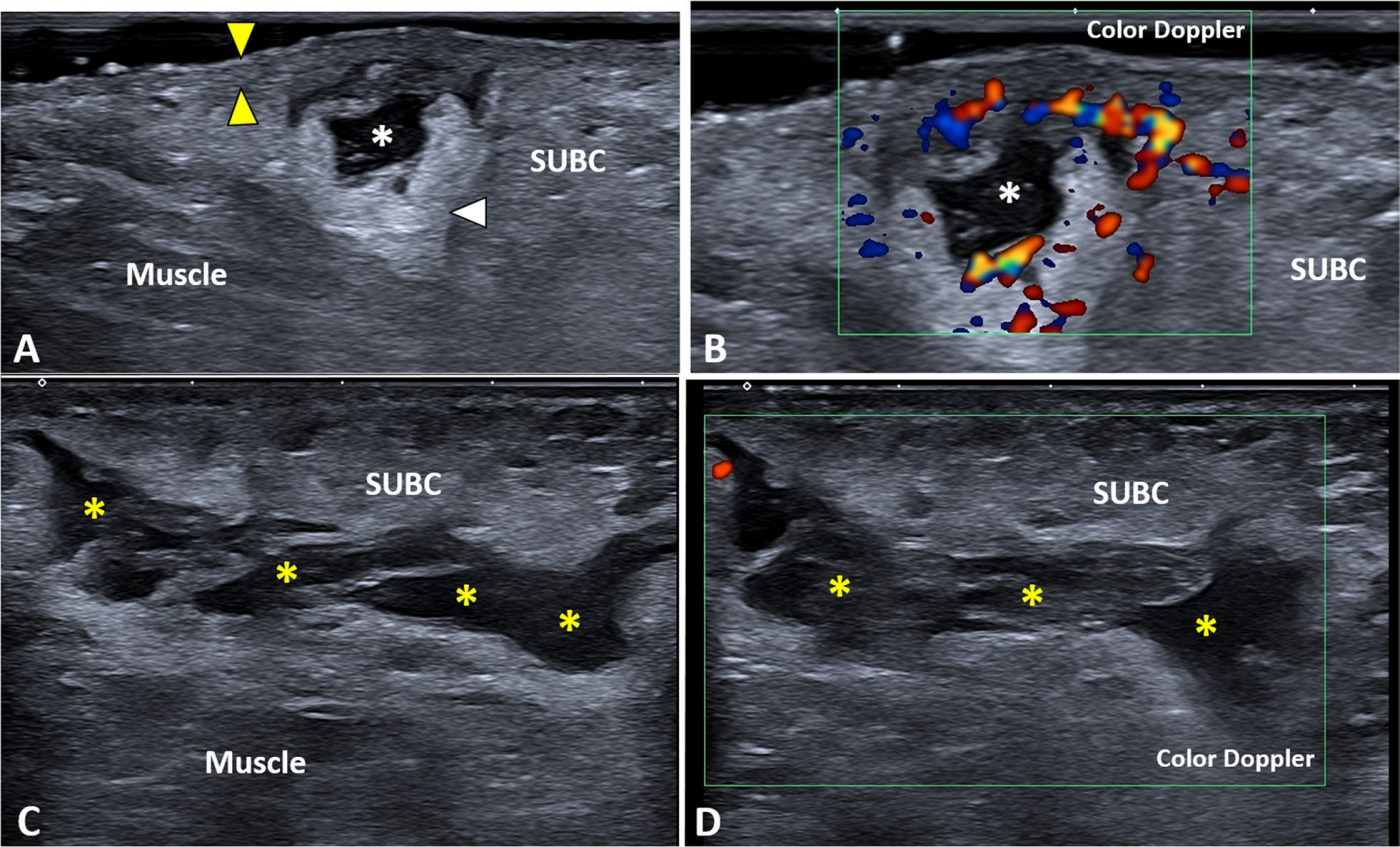
Subcutaneous abscess vs. hematoma of the axilla. A small, irregular-in-shape, fluid collection *(white asterisk)* can be identified within the axillary subcutaneous tissue *(SUBC)* not extending to the overlying dermo-epidermal complex *(yellow arrowheads)* nor to the underlying muscle planes (**A**); and high-sensitivity color Doppler shows intense hypervascularization of the surrounding soft tissues confirming inflammatory/infectious local phenomena (**B**). Of note, axillary hematoma *(yellow asterisks)* can present a sonographic pattern mimicking an abscess (**C**), but vascular signals are usually absent within the surrounding fat pad (**D**). White arrowhead: posterior acoustic enhancement

The subcutaneous abscess usually presents as irregular-in-shape fluid collection with a posterior acoustic enhancement (Additional file 6: Video 6) and aberrant hypervascularization in the periphery of the lesion (Additional file 7: Video 7) [26]. The latter sonographic finding is often the only difference between an abscess and hematoma (Fig. 8). We suggest performing a wide sonographic tracking of the axillary region in both the aforementioned pathological conditions in order to panoramically quantify the spatial extension of inflammatory fluid or blood (Additional file 8: Video 8). A gentle sono-palpation can be promptly performed to assess the mechanical deformability of the abscess/hematoma, differentiating it from irregular-in-shape solid masses.

Axillary lymphedema, like in other anatomical regions, usually presents a cobblestone pattern related to the progressive dilatation of the lymphovascular branches located within the fibrous scaffold of the subcutaneous tissue [4, 27]. Sonographic findings alone are usually not specific enough to differentiate primary vs secondary lymphedema; in this sense, a comprehensive assessment of the patient is necessary to optimize the diagnostic pathway. Primary lymphedema (i.e., lymphatic malformation) can be considered a heterogeneous cluster of lymphatic disorders involving the aplasia, hypoplasia and hyperplasia of lymphatic channels as well as the localized unifocal lesions characterized by dilated collectors partially disconnected from the main lymphovascular network [28]. Secondary lymphedema is instead often related to surgical resection of lymph nodes, disruption and obliteration of the lymphatic network due to radiotherapy, infection of the soft tissues, autoimmune diseases and local trauma [29].

Fat necrosis of the axilla commonly occurs postoperatively or after radiation therapy. On ultrasound, necrosis of the axillary fat pad shows variable patterns ranging from a solid hypoechoic mass with posterior acoustic shadow to a complex mass with heterogeneous inner echotexture and no vascularity on color/power Doppler [30].

Last but not least, among the several potential causes of iatrogenic injuries of the axillary fat tissue, postoperative fluid collections are quite common in clinical practice [14]. In the early phase, they usually show an anechoic appearance with a thin wall; likewise, in the chronic stage, a progressive thickening of the fibrous coat is coupled with the development of internal septa and floating echogenic deposits related to fibrinous debris—i.e., a pseudocystic collection. Of note, hyperechoic echoes within the anechoic fluid can also be related to different pathological conditions such as gas microbubbles due to local infection, inflammatory clots of white cells, hemorrhagic clots of red cells and foreign bodies; so, the differential diagnosis combining clinical and sonographic findings is paramount to correctly plan the management of patients [31].

### Lymph nodes

Sonographic assessment of lymph nodes requires a step-by-step evaluation of multiple morphological parameters; in this sense, the authors suggest in daily practice to strictly refer to a standardized checklist (Table 2) [31]. The shape of a lymph node is usually correlated with the ratio between the longitudinal and transverse diameter (L/T ratio). In this regard, a flattened lymph node with L/T > 2 is often identified in benign lymphadenopathy (Additional file 9: Video 9); instead, a rounded lymph node with L/T < 2 can be related to a malignant condition (Additional file 10: Video 10) [32].

**Table 2:** Sonographic assessment of lymph nodes

Parameters	Benign lymphadenopathy	Malignant lymphadenopathy
Shape	Flattened, oval (L/T r > 2)	Rounded, globular (L/T r < 2)
Hilum	Well defined	Blurred or absent
Cortex	Concentric thickening	Concentric or eccentric thickening Additional findings ^#^
Microvasculature	Unipolar vascularization	Multipolar vascularization Absent vascularization

^#^Focal areas of necrosis, calcific depositions, extracapsular invasion

**Fig. 9:**
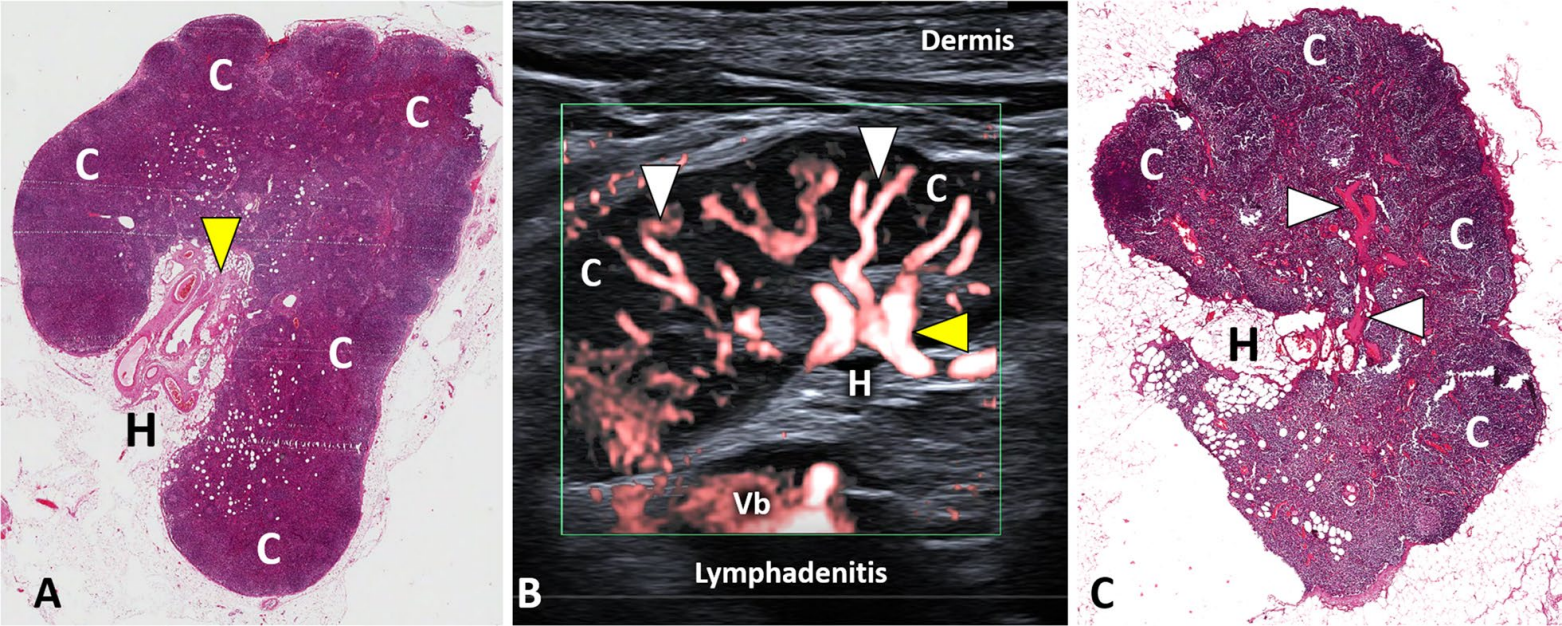
Sono-histological architecture of lymph nodes. Histological samples of reactive lymph nodes (**A, C**) clearly show how the feeding microvasculature *(yellow arrowhead)* enters through the hilum *(H)* to progressively branch smaller ramifications *(white arrowheads)* toward the cortex (**C**)—i.e., the unipolar vascularization. Of note, all the aforementioned histological elements can be easily identified using modern high-frequency probes and high-sensitivity color/power Doppler (**B**). Vb: vascular bundle; H&E: hematoxylin & eosin

**Fig. 10:**
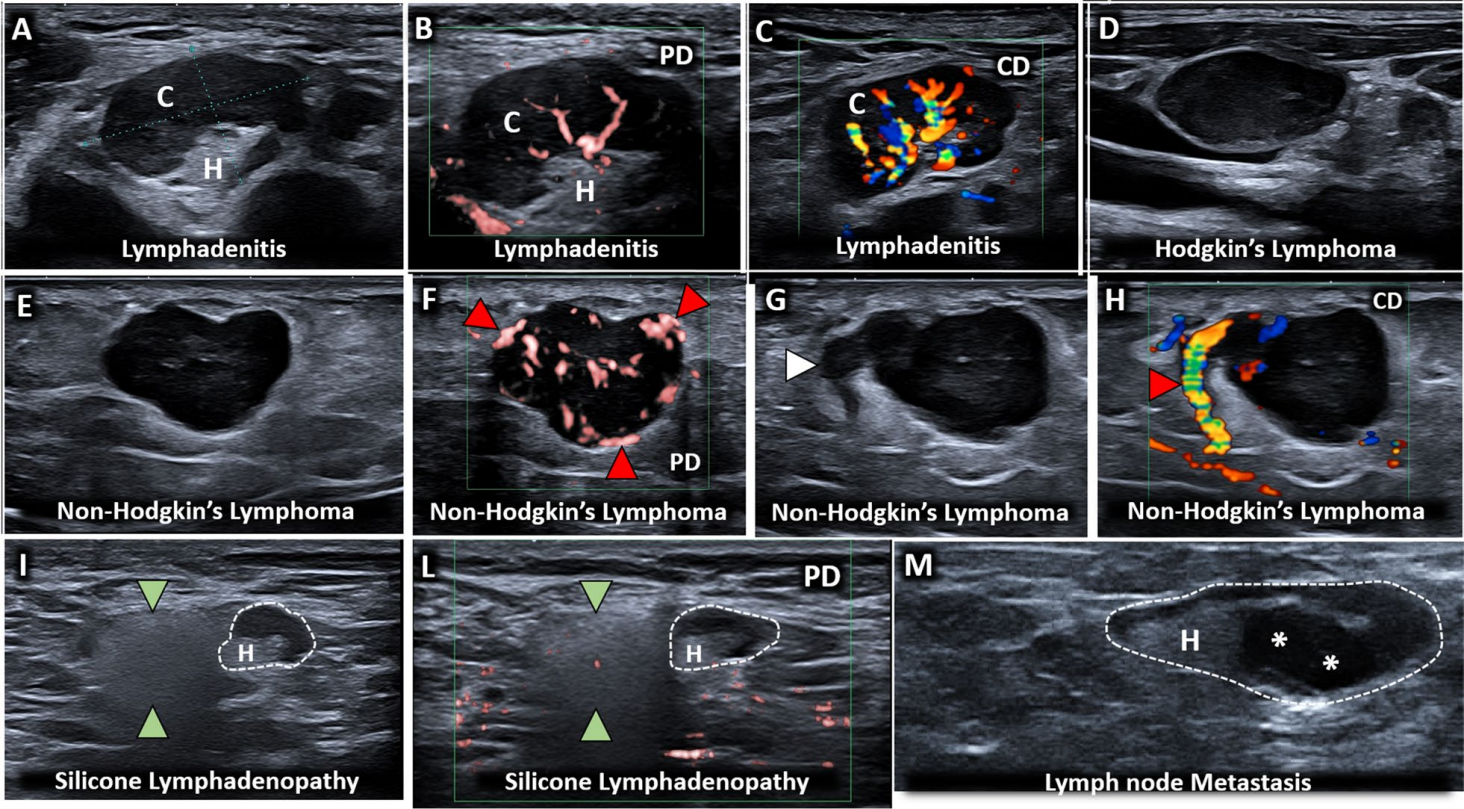
A glance-in-depth at the multiparametric sonographic assessment of lymph nodes. Benign lymphadenopathy (**A**) usually preserves the hyperechoic hilum *(H)*, the hypoechoic cortex *(C)* and a unipolar vascularization with feeding vessels branching from the hilum to the cortex (**B, C**) Instead, a disappearance of the hilum (**D**), a rounded/globular shape (**E**) and a multipolar vascularization (**F**) with feeding vessels *(red arrowheads)* originating from the peripheral portion, can be considered the most common sonographic findings of malignant lymphadenopathy. Of note, considering the extreme variability of sonographic patterns of lymph nodes, several “atypical” findings can be identified in daily practice—e.g., serpiginous protrusions *(white arrowhead)* related to ectatic vessels *(red arrowhead)* (**G, H**) a normal lymph node *(white dotted line)* coupled with a “snowfall” lymph node *(green arrowheads)* due to particle depositions (**I, L**) eccentric thickening of the cortex *(white asterisks)* with lateral displacement of the hilum *(H)* (**M**). PD: power doppler; CD: color doppler

The normal sonographic pattern of a lymph node presents a central hyperechoic area called the hilum and a peripheral hypoechoic portion—the cortex [32]. The high echogenicity of the hilum is related to the presence of multiple acoustic interfaces generated by arterial/venous vessels, lymphatic channels and fat tissue (Fig. 9) [32]. Of note, sclerolipomatosis is a frequent benign condition characterized by a progressive increase of the fat tissue surrounding the feeding vessels of the lymph node, and the corresponding sonographic pattern shows a large hyperechoic central area surrounded by a thin hypoechoic cortical tissue. Benign lymphadenopathy usually preserves the inner hyperechoic hilum, unlike malignant conditions where it is poorly visible or absent (Fig. 10) [31, 33]. Convex indentations of the fatty hilum (“rat bite” sign), concentric hilar compression and lateral displacement of the hilum have also been described in the pertinent literature as potential sonographic signs of malignancy [34]. Instead, as regards the cortical segment, angular edges coupled with the loss of the thin echogenic capsule enclosing the lymph node can be related to perinodal invasion in malignant pathologies [34].

In physiological conditions, color/power Doppler shows a unipolar perfusion pattern of the lymph node with flow signals entering through the hilum and regularly branching toward the peripheral portion [32]. Benign lymphadenopathy usually preserves the aforementioned spatial architecture of the microvasculature, but increases the amount/intensity of flow signals (Additional file 11: Video 11) [31–35]. On the other hand, malignant lymphadenopathy is characterized by a multipolar vascularization, anarchic distribution of the flow signals (Additional file 12: Video 12) and vessels at the level of the more cortical zones of the lymph node until the capsular coat (Additional file 13: Video 13) [33]. In fact, neoplastic cells reach the target lymph node passing through the afferent lymphatic channels and distribute with a peripheral to central progression inside it. The aforementioned “invasion mechanism” can be considered the main reason for the multipolar perfusion and subcapsular vascular signals commonly characterizing the malignant lymph nodes. The role of the resistive index has been described in the pertinent literature referring to lower vascular resistance in normal and inflammatory reactive lymph nodes compared to malignant ones [36]. Of note, also a completely different appearance with very poor or absent vascularization on color/power Doppler can characterize a malignant lymph node due to mechanical disruption of the microvasculature related to necrotic and/or fibrotic phenomena [33, 37].

It is paramount to keep in mind that focal sonographic abnormalities of the cortex of lymph node—e.g., nodular thickenings (Additional file 14: Video 14), calcific depositions, foci of necrosis—can characterize both benign and malignant lymphadenopathies; for this reason, their presence should be always integrated with the clinical findings. In this sense, considering the wide spectrum of non-neoplastic disorders presenting axillary adenopathy—mastitis, cellulitis, hidradenitis, local infections (e.g., tuberculosis, mononucleosis, cat scratch disease), autoimmune diseases (e.g., rheumatoid arthritis, systemic lupus erythematosus, psoriasis, scleroderma and dermatomyositis) and granulomatous diseases (e.g., sarcoidosis, tuberculosis and silicone-induced granulomatous adenitis) should be always considered in the differential diagnosis [38].

Elastography seems to be a promising diagnostic tool to optimize the differentiation between normal and pathological lymph nodes. For instance, Wojcinski et al. have demonstrated that the cortex of metastatic lymph nodes is significantly harder than the cortex of healthy lymph nodes, with a predominance of the colors blue and turquoise in the corresponding elastograms [39]. Contrast-enhanced ultrasound is an additional technique that—by using microbubbles smaller than red blood cells (1–4 µm)—allows visualization of changes in the microvasculature architecture of the lymph node and the eventual presence of avascular areas [40]. Interestingly, malignant lymph nodes may present inner areas not perfused by the contrast agent (i.e., hypoenhancing areas)—due to avascular necrotic depositions of neoplastic cells—defining the most suitable sites where to perform the ultrasound-guided biopsy [41]. Of note, the strictly intravascular distribution of the contrast agent also guarantees a follow-up of the intra-lymph node hypervascularization during the antineoplastic treatments [40, 41]. Lastly, contrast-enhanced ultrasound can also be considered a potential diagnostic tool to early identify focal, nodular neoplastic recurrence within fibrous scar tissue to accurately guide the biopsy [40, 41].

### Neurovascular bundle

At the axilla, the most common pathologies involving the neurovascular bundle in daily practice are arterial/venous thrombosis and neural benign neoplasms such as schwannoma.

**Fig. 11:**
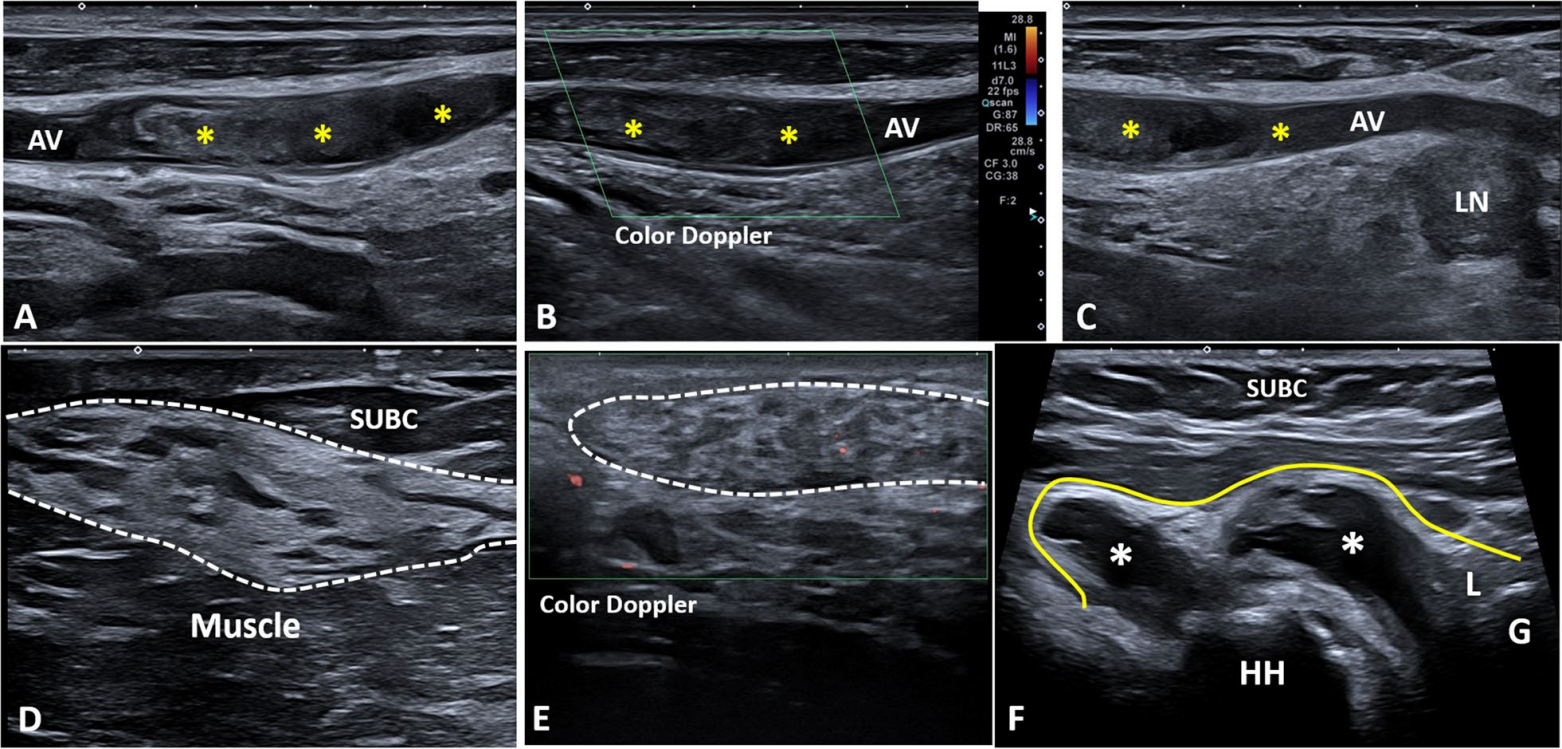
Additional sonographic findings of the axilla. Longitudinal scans (**A**–**C**) show a massive thrombosis *(yellow asterisks)* of the axillary vein *(AV)* coupled with a globular lymph node *(LN)* in a patient with neoplastic disease. Accessory breast tissue *(white dotted line)* is usually located in between the subcutis *(SUBC)* and muscle planes (**D**) showing a peculiar sonographic pattern with hyperechoic fibroductal matrix, hypoechoic fat islands and poor vascular signals in normal conditions (**E**). Articular effusion *(white asterisks)* distending the axillary recess *(yellow line)* can be easily identified using the standing position in a patient with glenohumeral osteoarthritis (**F**). *HH* Himeral head, *G* Glena, *L* Labrum

Endoluminal thrombotic material usually presents as echoic tissue located within the axillary artery/vein non-compressible during the sono-palpation. Longitudinal and transverse scans in grayscale modality should be coupled with the color/power Doppler assessment in each and every patient to confirm the absence of blood flow within the target vessel (Fig. 11) [42]. Considering the atypical location of thrombotic disease at this level, the authors suggest accurately scanning the lymph nodes wrapping the axillary vessels. In these patients, it is not infrequent to identify a paraneoplastic thrombosis of the axillary vascular bundle coupled with metastatic lymph nodes at this level (Additional file 15: Video 15) [43].

Schwannoma (neurilemoma) is a peripheral nerve sheath tumor originating from differentiated neoplastic Schwann cells. The sonographic pattern of the schwannoma is highly variable, but most commonly, it presents as a solid hypoechoic mass with well-defined edges and a slight posterior acoustic enhancement [44]. A longitudinal scan can be performed to promptly demonstrate the anatomical continuity of the neoplastic mass with the proximal and distal segments of the nerve from which it originates. Moreover—especially using modern high-frequency ultrasound probe—a gentle shifting/tilting of the probe allows the identification of structural continuity between the hypoechoic mass and a specific swollen fascicle, with a simultaneous spatial displacement of the normal fascicles toward the inner surface of the epineurial sheath [45]. The schwannoma remains strictly confined within the hyperechoic epineurium without extra-sheath dissemination of the neoplastic cells [45]. Of note, chronic schwannomas can progressively develop degenerative changes such as intraneural cysts, calcific depositions, hematomas and focal fibrosis [44]. High-sensitivity color/power Doppler can be used to accurately assess the perfusion pattern of the aforementioned neural mass—intrinsic and extrinsic microvasculature system [3].

### Additional sonographic findings

Accessory breast tissue can persist from embryological development up to 6% of the general population, and at the level of the axillary region, it is usually located along the so-called milk line. Histologically, it presents as subcutaneous fibroglandular tissue not in continuity with the main breast parenchyma. The sonographic pattern is usually characterized by a hyperechoic fibroductal scaffold with small-size hypoechoic fat lobules (Fig. 11) [12].

Adjusting the settings of the ultrasound machine (e.g., range of frequency, the position of the focal zone), the deepest section of the axilla can be assessed using linear probes—the inferior portion of the glenohumeral joint. Using the humeral head laterally and the glenoid of the scapula medially as bony landmarks, a longitudinal scan of the joint can be easily performed [17]. Distension of the axillary recess of the glenohumeral capsule (Additional file 16: Video 16) can be related to several shoulder pathologies, and the standing position allows the articular effusion to collect at this level due to gravity force (Fig. 11) [46]. Of note, considering the spatial arrangement of the glenohumeral synovial membrane, the axillary pouch and the anterior bicipital recess are the two most common anatomical sites of the shoulder where intra-articular loose bodies can be identified.

Interestingly at this level, in a longitudinal-oblique plane, the color/power Doppler can be used to promptly identify the posterior humeral circumflex artery encircling the humeral head and slightly swaying the probe, also the axillary nerve can be promptly visualized coursing around the inferior glenohumeral joint capsule with the classical fascicular pattern (Additional file 17: Video 17) [47]. Likewise, by moving the transducer more laterally, the axillary nerve in the long axis can be traced within the fascial plane between the long head of triceps and deltoid muscle [47].

**Fig. 12:**
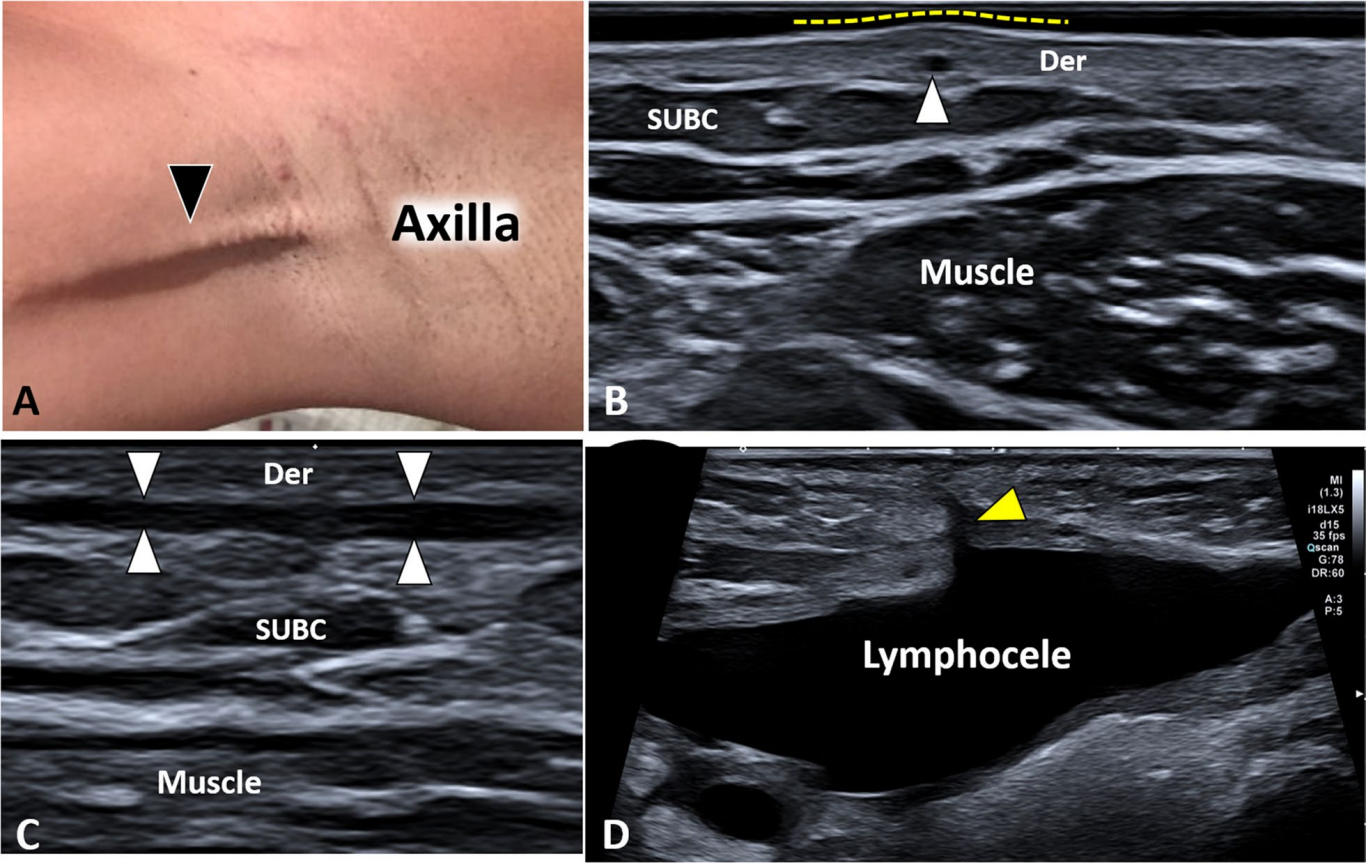
Axillary web syndrome and lymphocele. Axillary web syndrome (**A**) is clinically characterized by very superficial, painful and retractile cords *(black arrowhead)*. Transverse (**B**) and longitudinal (**C**) sonogram—using a high-frequency probe—shows the hypoechoic band *(white arrowheads)* coursing within the dermal layer *(Der)* of the axillary region. Of note, a large amount of gel allows a prompt identification of the skin bulging *(yellow dotted line)* where the lymphatic cord *(white arrowhead)* should be sonographically assessed (**B**). A large lymphocele (**D**) presents as anechoic irregular-in-shape fluid collection and its peduncular connection *(yellow arrowhead)* with the overlying surgical incision is clearly visible as well. SUBC: subcutaneous tissue

Peculiar lymphatic diseases, especially after surgical resection of axillary lymph nodes, can be identified at this level, and among the others, axillary web syndrome (AWS) and lymphocele are the most frequent [27]. The histopathological findings seem to define the AWS as thrombosis of superficial lymphatic channels with the development of painful and retractive “cords” [48]. On ultrasound, the cords are usually located within the dermal layer and they present as hypoechoic thin bands with hyperechoic edges (Fig. 12) [49]. Of note, using a large amount of gel can be considered a simple technical tip to promptly localize the “bulging” of the skin surface where the cord is located. Lymphocele is a collection of lymphatic fluid not bordered by epithelial lining, usually related to extensive lymphadenectomy. The sonographic pattern can be highly variable, ranging from a simple anechoic fluid collection (Fig. 12) to a “complex mass” with heterogeneous texture and multiple inner septa (Additional file 18: Video 18) [27, 50]. The latter appearance is usually related to the high concentration of proteins in suspension within the lymphatic fluid that progressively lead to a fibrotic organization of the lymphocele.

### Pitfalls and limitations

The lymph nodes can be considered the most challenging anatomical elements to sonographically assess in the axillary region; indeed, while matching multiple morphological parameters, several “gray zones” of differential diagnosis between benign and malignant lymphadenopathy are still the subject of debate. In this sense, we have reported some technical aspects aiming to avoid unnecessary misinterpretation of ultrasound images in clinical practice.

Axillary lymph nodes usually present a less bright hilum compared to other anatomical sites, and this site-related variation should not be misinterpreted as a pathological finding; moreover, in very small (normal) lymph nodes, it is not always clearly visible hyperechoic hilum.

In the advanced phases of infectious diseases, lymph nodes can develop focal necrosis and colliquative phenomena with marked disorganization of the histological architecture acquiring a globular shape similar to malignant lymphadenopathies. Moreover, calcific depositions within the lymphoid tissue, skin fistulizations and extracapsular abscesses can be related to specific infectious lymphadenopathy, further complicating the differential diagnosis.

Perfusion pattern of low-grade lymphomas can be quite similar to that of reactive lymphadenitis (lymphoid hyperplasia) with a saving of the cortical zone and hypertrophy of the vessels in the hilum (Additional file 19: Video 19); in this sense, all the sonographic parameters must be accurately coupled with a medical history and physical examination. Granulomatous lymphadenopathy can show a very poor vascularization on color/power Doppler—due to fibrotic/necrotic evolution of the lymphoid tissue—mimicking a malignant lymph node.

## Conclusion

Axilla is a complex anatomical region, and to the best of our knowledge, a standardized layer-by-layer sonographic approach is lacking. A step-by-step comprehensive sonographic approach, including pathological sonographic findings with the corresponding histopathological features—i.e., the so-called sono-histological patterns, is crucial for the detection and characterization of axillary diseases. This ultrasound approach could be considered a ready-to-use educational guidance for the assessment of the axillary region.

## Supplementary Information

**Additional file 1**. Epidermal inclusion cyst presents as a rounded anechoic mass simultaneously involving the dermis and subcutaneous tissue.

**Additional file 2**. High-sensitivity color Doppler shows vascular signals within an axillary pseudocyst in a patient with hidradenitis suppurativa.

**Additional file 3**. The very high-frequency ultrasound probe clearly shows a saclike fluid collection—within the dermis of the axillary region—in a patient with hidradenitis suppurativa.

**Additional file 4**. High-sensitivity color Doppler promptly shows intense vascular signals within the hypoechoic thickening of the dermis in a patient with axillary hidradenitis suppurativa.

**Additional file 5**. Using a large amount of gel—i.e., the suspension technique—a hypoechoic oval lesion can be easily identified in the dermis. Of note, the inner “striped” pattern is related to the presence of hyperechoic hairs.

**Additional file 6**. Subcutaneous abscess commonly presents as an irregular, hypo/anechoic fluid collection located deeper than the dermal layer. Of note, a well-defined capsular wall is not clearly visible and the edges are blurred.

**Additional file 7**. High-sensitivity color Doppler promptly shows intense hypervascularization of the soft tissues surrounding the collection. The latter can be considered a pivotal technical aspect in the differential diagnosis of abscess vs. hematoma.

**Additional file 8**. A wide sonographic tracking—from pectoralis major muscle anteriorly to latissimus dorsi muscle posteriorly—promptly shows the spatial distribution of post-traumatic axillary hematoma.

**Additional file 9**. A flattened, axillary lymph node, with an L/T ratio >2, can be easily identified in a patient with reactive lymphadenitis. Of note, the hyperechoic hilum is clearly recognizable as well.

**Additional file 10**. A globular lymph node, with an L/T ratio <2, can be visualized in the axillary region of a patient with non-Hodgkin’s lymphoma. Of note, the hyperechoic hilum is not visible.

**Additional file 11**. High-sensitivity color Doppler shows an intense hypervascularization of an axillary lymph node related to post-vaccine lymphadenitis. Of note, the unipolar perfusion pattern is completely preserved, with a microvasculature branching from the hilum.

**Additional file 12**. High-sensitivity color Doppler promptly shows the irregular perfusion pattern of an axillary lymph node in a patient with non-Hodgkin’s lymphoma. Of note, the hilum is not visible and multiple flow signals are randomly distributed within the cortex.

**Additional file 13**. High-sensitivity color Doppler precisely displays the subcapsular hypervascularization of an axillary lymph node in a patient with Hodgkin’s lymphoma. Of note, the hyperechoic hilum is absent.

**Additional file 14**. Ultrasound-guided biopsy of a focal nodular thickening selectively involving the peripheral portion of an axillary lymph node in a patient with melanoma. Of note, the cortical mass displaces the hyperechoic hilum in an eccentric position.

**Additional file 15**. A longitudinal scan of the axillary region clearly shows the thrombosis of the axillary vein and a large metastatic lymph node in the same patient with a previous diagnosis of neoplastic disease.

**Additional file 16**. Adjusting the technical settings of the ultrasound machine to “explore” the deepest portion of the axillary region, articular effusion distending the axillary pouch can be easily observed in a patient with glenohumeral osteoarthritis.

**Additional file 17**. Longitudinal oblique scan of the axillary artery/nerve at the level of axillary fold. Of note, the fascicular pattern of the nerve can be nicely identified beneath the pulsating artery.

**Additional file 18**. A chronic lymphocele of the axillary region appears as a rounded “complex mass” with heterogeneous echotexture alternating hypo/anechoic foci with hyperechoic septa and fibrotic tissue.

**Additional file 19**. In this patient, at high-sensitivity color Doppler, a low-grade non-Hodgkin’s lymphoma presents hypertrophic vasculature at the hilum and poor flow signals of the peripheral lymphoid tissue. Of note, the aforementioned perfusion pattern can be commonly identified also in post-infectious lymphadenitis.

## Data Availability

Not applicable.

## Abbreviation

AWS: Axillary web syndrome
